# Co-evolution of quaternary organization and novel RNA tertiary interactions revealed in the crystal structure of a bacterial protein–RNA toxin–antitoxin system

**DOI:** 10.1093/nar/gkv868

**Published:** 2015-09-08

**Authors:** Feng Rao, Francesca L. Short, Jarrod E. Voss, Tim R. Blower, Anastasia L. Orme, Tom E. Whittaker, Ben F. Luisi, George P. C. Salmond

**Affiliations:** Department of Biochemistry, University of Cambridge, Cambridge CB2 1QW, UK

## Abstract

Genes encoding toxin–antitoxin (TA) systems are near ubiquitous in bacterial genomes and they play key roles in important aspects of bacterial physiology, including genomic stability, formation of persister cells under antibiotic stress, and resistance to phage infection. The CptIN locus from *Eubacterium rectale* is a member of the recently-discovered Type III class of TA systems, defined by a protein toxin suppressed by direct interaction with a structured RNA antitoxin. Here, we present the crystal structure of the CptIN protein–RNA complex to 2.2 Å resolution. The structure reveals a new heterotetrameric quaternary organization for the Type III TA class, and the RNA antitoxin bears a novel structural feature of an extended A-twist motif within the pseudoknot fold. The retention of a conserved ribonuclease active site as well as traits normally associated with TA systems, such as plasmid maintenance, implicates a wider functional role for Type III TA systems. We present evidence for the co-variation of the Type III component pair, highlighting a distinctive evolutionary process in which an enzyme and its substrate co-evolve.

## INTRODUCTION

Bacterial toxin–antitoxin (TA) systems were discovered nearly 30 years ago in studies of plasmid stabilization during cell division (1,2). Genes encoding TA systems are now known to be very widespread in prokaryotic genomes (3). TA systems have been classified into five types. All share common features in that they include a toxic protein component and are usually bicistronic in architecture, but are distinguished from each other by the manner in which this toxic protein is antagonized. In Type I systems, the antitoxin is an antisense RNA molecule that prevents translation of what is usually a small hydrophobic toxin. In Type II systems the antitoxin is a co-expressed protein that directly inhibits the toxin. Type IV and V TA systems also utilize protein antitoxins; Type IV TA components do not physically interact and in the prototype system both proteins act in opposing manners on a common substrate (4,5). The single described Type V TA system employs a ribonuclease antitoxin that targets the toxin transcript (6). The focus of the present study, Type III systems, comprise a ribonuclease (RNase) toxin that processes, and is then directly inhibited by, its structured and specific cognate RNA antitoxin.

Recent advances in sequencing technology and the associated exponential increase in available genomic data have enabled large-scale bioinformatic surveys of TA loci, aimed both at finding locations of known families and discovering new TA families within the many open reading frames (ORFs) of unknown function (7,8). As it became clear that TA system loci are near-ubiquitous in bacterial genomes (and often in very high numbers within a single species) the definition of their role(s) in bacterial physiology became a more pressing issue. TA systems have been associated with multiple important phenotypes, including phage resistance, maintenance of genomic islands and formation of bacterial persister cells (9). The widespread distribution and redundancy of the TA systems, especially in the Type II group, suggests that TA pairs are important for organism fitness (10–12).

The paradigmatic Type III TA locus, *toxIN*_Pa_, was discovered on a cryptic plasmid of *Pectobacterium atrosepticum* strain SCRI1039. A gene on this plasmid encoded a homologue of a known phage resistance protein, and this provided strong protection from some bacteriophages while also being extremely toxic to the cell (13). This toxic gene could only be cloned in concert with short tandem repeats located upstream; these repeats were shown to encode a small RNA that suppressed the activity of the toxin. The components were named ToxN_Pa_ and ToxI_Pa_ (for Toxin Inhibitor). Structural studies of *toxIN*_Pa_ revealed that the ToxN_Pa_ ribonuclease cleaved the repetitive ToxI_Pa_ RNA into individual 36 base pseudoknot repeats, and then assembled with these catalytic products into a heterohexameric, triangular complex that inhibits the toxin (14). Interestingly, ToxN_Pa_ was shown to be a distant structural homologue of the Kid family of Type II TA system toxins.

The corresponding ToxIN_Bt_ complex from *Bacillus thuringiensis* showed a largely similar architecture to that of *P. atrosepticum*, but with subtle structural changes that underpinned highly specific TA recognition between cognate partners (15). While the heterohexameric organization of three ToxNs and three ToxIs was retained in this homologue, the interface between the RNA and protein showed key changes in the variable loops outside of the structural core of the toxin that allow the selective recognition of its cognate antitoxic RNA.

Over 120 putative Type III TA loci have been identified bioinformatically, in very diverse bacterial species, and these systems fall into three separate families (named ToxIN, CptIN and TenpIN) based on sequence similarity of their toxin components (16). While it is presumed that each locus contains a structured RNA as its functional antitoxin, as in the *toxIN*_Pa_ paradigm, the tandem repeats differ greatly in both primary sequence and length. Members of all three families have been shown to function as TA loci, and at least one other family (the TenpINs) can also mediate phage resistance (16).

It has also become increasingly clear that RNA species play a pivotal role in controlling gene expression in bacteria (17). Since the realization of the importance of Hfq as a regulator through its function as an RNA chaperone (18) as well as the discovery of riboswitches (19), RNA is no longer seen as a passive informational mediator. Similarly, the discovery of systems of acquired phage immunity (CRISPR) is another development that illuminates the ways in which cellular proteins are able to directly use the information contained within RNA species (20). While RNAs have been postulated to be natural mediators of protein activity, based on the precedent of artificial aptamers (21), most known examples show the RNA mimicking DNA (17). The distinctive case in which ToxN is specifically regulated by a pseudoknot RNA species which the toxin itself processes is a unique example in the growing repertoire of riboregulation (14).

Here, we present the first structural investigation of a member of the CptIN family of Type III TA systems. Many Type III TA systems are plasmid-encoded (16) but we decided to investigate the chromosomally-encoded CptIN system, from the human commensal *Eubacterium rectale* (CptIN_Er_) as the first representative of the CptIN family. CptIN_Er_ forms a dramatically different complex from that of the previously described ToxIN systems, exhibiting a unique oligomeric structure. We demonstrate that, while CptI_Er_ retains the pseudoknot core previously observed in other Type III antitoxins, the extended length of the RNA leads to the formation of unique tertiary features. However, the overall principles of toxin inhibition are shared between the CptIN and ToxIN families. The present study reveals the structural diversity that can exist in these loci and highlights the remarkable case in which an enzyme co-evolves with its cognate substrate.

## MATERIALS AND METHODS

### CptIN_Er_ purification, crystallization and structure determination

The ORF encoding CptN_Er_ was cloned into pTYB1 (NEB) using primers TRB273 and TRB274 (Table 2), with a three-amino acid extension (LEG) to enable intein cleavage. The CptI_Er_ gene was cloned into pACYC184 (22) along with a T7 promoter 3′ of the predicted start of the gene, identical to that present in pTYB1, using the primers TRB271 and TRB272 (Table 2). The resulting plasmids were introduced to *Escherichia coli* ER2566 (NEB) by electroporation. For native protein expression a 500 ml culture in 2×TY media was inoculated with an overnight culture of the expression strain at a 1:100 dilution and was grown at 37°C to OD_600_ 0.8. The cells were then shifted to 18°C, expression of CptIN_Er_ was induced with a final concentration of 1 mM IPTG, and expression was continued for 16 h. Expression of the SeMet derivative was performed using a supplemented minimal medium, as described (23). The cells were resuspended in 20 mM Na_2_HPO_4_, 500 mM NaCl, 1% v/v Triton X-100, 1 mM ethylenediaminetetraacetic acid, pH 6.0 and lysed by pressure with four passes through an EmulsiFlex-C5 instrument at 10 000 psi. The CptIN_Er_ protein–RNA complex was purified initially by passing the clarified lysate through a chitin column followed by on-column cleavage of the intein fragment fused to the CptN_Er_ protein under reducing alkaline conditions. The eluate from the cleavage step was then fractionated by anion exchange chromatography using a HiTrap Q 5 ml column (GE Healthcare), yielding one sharp peak identified as the CptIN_Er_ complex. The protein–RNA complex was then concentrated to ∼5 mg.ml^−1^ and used in crystallization trials.

Initial crystallization trials were carried out in 96-well tray sitting drops of 200 nl of protein mixed with 200 nl of precipitant in a vapour diffusion system with 200 μl of the precipitant in a reservoir, using the Classics, JCSG+ and Protein Complex commercial screens (Qiagen). Hits were optimized using 24-well tray hanging drops of 1 μl of protein mixed with 1 μl of precipitant with 500 μl of precipitant in the reservoir. The best diffracting crystals for both the native and the SeMet derivative were from a condition containing 200 mM CaCl_2_, 100 mM CH_3_CO_2_Na, 20% v/v 2-propanol, pH 4.8. Data collection was performed on the I-02 beamline at the Diamond Light Source synchrotron facility (Harwell, Oxford, UK), with a single crystal of the native complex diffracting to 2.2 Å and two crystals of the SeMet derivative, both of which diffracted to 3.0 Å (summarized in Table 1). Data processing and reduction were performed on these datasets using iMOSFLM (24), SCALA (25) and TRUNCATE (26) from the CCP4 suite of programs (27). The combined SeMet data was input as a single-wavelength anomalous dispersion (SAD) set into the AutoSolve function in PHENIX (28), yielding a solution with 40 selenium sites (corresponding to eight protein monomers) with a Figure of Merit of 0.385 and a readily interpretable electron density map. It was noticed that where there was obvious density corresponding to RNA, protein residues had instead been placed by PHENIX. For this reason, the prebuilt chains were removed and chain re-traced using firstly BUCCANEER (29) for the protein and then NAUTILUS (30) for the RNA. Manual chain building was continued using COOT (31), as well as the manual addition of waters; very high positive peaks observed in the F_O_-F_C_ map showed the presence of Ca^2+^ ions from the crystallization condition, also corroborated by the presence of clear octahedral coordination from adjacent waters and RNA phosphate groups. The model could then be directly refined using REFMAC5 (32) against the isomorphous native dataset, and the final 2.2 Å structure contained eight protein chains of CptN_Er_ from residues 1 to 155, as well as eight full 45 nt chains of the CptI_Er_ RNA (with Ramachandran statistics of 98.3% preferred, 1.6% allowed and 0.1% outliers).

**Table 1.** Crystallographic and structural refinement data

	Native	SeMet
**Diffraction Statistics**		
Space group	P2_1_	P2_1_
*Cell dimensions*		
a, b, c (Å)	63.1, 185.9, 138.8	63.2, 186.5, 139.2
α, β, γ (°)	90, 92.63, 90	90, 92.58, 90
Resolution (Å)	2.2 (2.24–2.20)	3.0 (3.07–3.00)
Rmerge	0.048 (0.338)	0.164 (0.612)
I/σI	11.6 (2.4)	13.9. (4.8)
Completeness (%)	97.9 (97.0)	100 (100)
Redundancy	2.3 (2.1)	13.5 (12.6)
Number of unique reflections	157 960 (7761)	64 337 (4487)
Wilson B factor (Å^2^)	28.8	44.1

**Refinement statistics**		
Rfactor	0.20	
Rfree	0.23	
Number of reflections used	161 398	
*Number of atoms*		
Protein	10409	
RNA	7696	
Ion	52	
Water	1330	
*Average B factors*		
Protein	41.4	
RNA	42.6	
Ion	59.9	
Water	39.8	
Rmsd (bonds)	0.013	
Rmsd (angles)	1.72	

### RNA structural searches

AMIGOS II (33) was used to calculate RNA torsional angles and to perform structural searches. Structural searches were performed using the ‘Worm search’ function on all available RNA structures in the PDB.

### Construction of CptI_Er_ mutants

Overexpression vectors encoding either a single CptI_Er_ repeat, or a mutated repeat, were constructed using a strategy described previously (13), where a PCR was performed with pQE-80L as the template, using PF185 together with a reverse primer encoding the repeat sequence. An additional 5′-GA and 3′-AAG was included at the boundaries of the repeat to allow processing by CptN_Er­_. Primer and plasmid details are in Table 2.

**Table 2.** Strains, plasmids and primers used in this study

Strain	Genotype	Reference
*Escherichia coli* DH5α	F^−^ λ^−^*endA1 glnV44* t*hi-1 recA1 relA1 gyrA96 deoR nupG* Φ80 lacZ ΔM15 Δ(*lacZYA-argF*) U169, *hsdR17*(r_K_^−^ m_K_^+^)	Invitrogen
*E. coli* ER2566	F^−^ λ^−^*fhuA2* [*lon*] *ompT lacZ*::T7 *gene 1 gal sulA11* Δ(*mcrC-mrr*)114::IS10 R(*mcr-73*::miniTn*10*-Tet^S^)2 R(*zgb-210*::Tn*10*)(Tet^S^) *endA1* [*dcm*]	NEB
*E. coli* W3110	F^−^ λ^−^*rph-1* INV(*rrnD, rrnE*)	(39)
		
**Plasmid**	**Description**	**Source**
pALO3	*cptN*_Er_ in pTYB1	This study
pALO5	*cptI*_Er_ with P_T7_ upstream in pACYC184	This study
pFLS104	*cptI*_Rt_ repeats and terminator in pTA100, Sp^R^	(16)
pFLS105	*cptN*_Rt_ in pBAD30, Ap^R^	(16)
pFLS106	*cptI*_Cc_ repeats and terminator in pTA100, Sp^R^	(16)
pFLS107	*cptN*_Cc_ in pBAD30, Ap^R^	(16)
pFLS108	*cptI*_Er_ repeats and terminator in pTA100, Sp^R^	(16)
pFLS109	*cptN*_Er_ in pBAD30, Ap^R^	(16)
pFLS110	*cptI*_Er_ single repeat in pTA100, Sp^R^	This study
pFLS111	*cptI*_Er_ ΔU30 single repeat in pTA100, Sp^R^	This study
pFLS112	­*cptI*_Er_ A29U single repeat in pTA100, Sp^R^	This study
pFLS122	*cptIN*_Er_ locus in pRBJ200, Ap^R^	This study
pRBJ200	*E. coli par*-deficient single-copy vector, Ap^R^	(40)
pTA100	Sp^R^ derivative of pQE-80L	(13)
		
**Primer**	Sequence 5′-3prime;* (Restriction site)	
CptI-WT	tttaagcttTCAACCCGACCATTTATATACCACATATCGGTCAGTGGTAAACTTtcaattgaatctattataattgtta (HindIII)	
CptI-dU30	tttaagcttTCAACCCGACCATTTTATACCACATATCGGTCAGTGGTAAACTTtcaattgaatctattataattgtta (HindIII)	
CptI-A29U	ttaagcttTCAACCCGACCATTTAAATACCACATATCGGTCAGTGGTAAACTTtcaattgaatctattataattgtta (HindIII)	
PF185	AAACAAATAGGGGTTCCG	
TRB271	TTGGATCCATAATCAGTATCACTGAGAAA (BamHI	
TRB272	TTTCTAGAACTACGGCAAAAGACTTTTTC (XbaI)	
TRB273	CCGGCATATGATAAGGAATGGTTTTTATATTATC (NdeI)	
TRB274	GTGGTTGCTCTTCCGCACAAATGTGTTGCTGGTAAATC (SapI)	

*For CptI primers, the region encoding the repeat is shown in uppercase.

### CptI antitoxicity and cross-inhibition assays

Antitoxicity assays were performed in *E. coli* DH5α that had been transformed with separately-inducible CptI and CptN plasmids (Table 2). Assays were performed as described previously (13,16).

### Plasmid loss assays

Plasmid loss assays were performed in *E. coli* W3110 carrying either pFLS122 or pRBJ200 (Table 2). Experiments were performed essentially as described (34), with non-selective exponential growth maintained for 24 h.

## RESULTS

### CptIN_Er_ is a heterotetramer

**Figure 1.**
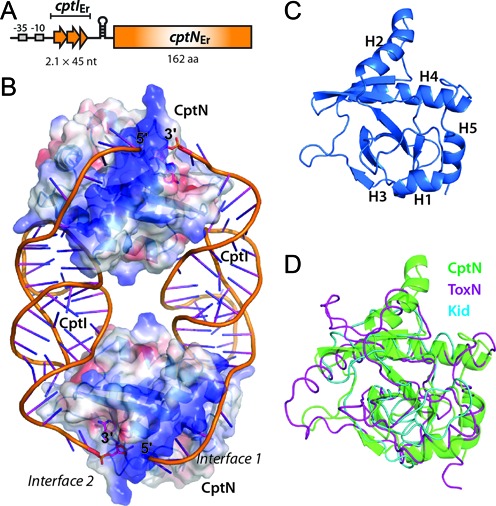
The crystal structure of CptIN_Er_ to 2.2Å resolution. (**A**) The architecture of the CptIN_Er_ locus, showing the tandem repeats upstream of the toxic CptN_Er_ ORF that become processed into CptI_Er_ monomers. (**B**) When analysed with symmetry cells in conjunction with data from size exclusion chromatography, the biological unit was found to be a heterotetramer—an oval structure with two protein monomers at the poles (CptN_Er_) held together by two pseudoknotted RNAs (CptI_Er_). The surface representation shows the electrostatic potential (red: electronegative, blue: electropositive. (PDB: 4RMO) (**C**) The structural core of the protein is a highly twisted β-sheet surrounded by α-helices. (**D**) The overlay with carbon-α traces of ToxN_Pa_ (magenta, PDB: 2XDD) and Kid, a Type II RNase (cyan, PDB: 1M1F), shows the lack of the kinked helix seen in ToxN_Pa_ in CptN_Er_, but still an overall similarity in fold.

The *cptIN* locus of *E. rectale* (Figure 1A) was previously shown to have TA function (16). To explore the structural basis for this activity, and the extent to which its mechanism is shared with ToxIN systems, the structure of the CptIN_Er_ complex was solved by X-ray crystallography. CptI_Er_ co-purified with the CptN_Er_ protein following overexpression in *E. coli*, and the stable protein–RNA complex was crystallized in both the native and SeMet forms. The best diffracting crystals (1 native and 2 SeMet) were isomorphous, and, following phasing through SAD, the final structure was solved to a resolution of 2.2 Å (Table 1).

The unit cell contained a total of eight protein (CptN_Er_) monomers each complexed with a single CptI_Er_ RNA repeat of 45 nt. Examination of the symmetry units (Supplementary Figure S1) reveals that each protein–RNA heterodimer formed a self-closing, heterotetrameric ring-shaped complex with a heterodimer from the neighbouring asymmetric unit (Figure 1B). The idea that the biological CptIN complex was the heterotetramer (of two protein monomers and two RNA ligands) observed in the crystal structure was corroborated by analytical size exclusion chromatography (Supplementary Figure S2 and Table S1). Note that both crystallization and size exclusion chromatography use high concentrations of CptN and CptI; the concentration of these molecules in the cell is unknown, and complex assembly may be strongly influenced by the turnover of these components and their interactions with other molecules. Studies on a homologous system found that the results from gel filtration could be further corroborated by analytical ultracentrifugation. The heterotetrameric architecture is a major deviation from the heterohexameric complexes formed by the other structurally-defined Type III TA systems.

The structural core of the CptN protein is similar to the structures of the three previously solved Type III toxins, being made up of a highly twisted antiparallel β-sheet (Figure 1C). This is surrounded by four α-helices, which, along with the many loops in the structure, make extensive contacts with the cognate RNA (Figure 1B). Of note is the absence of the kink in the third helix that is seen in the structures of ToxN_Pa_ (14), ToxN_Bt_ (15) and AbiQ (35); the equivalent helix H4 in CptN_Er_ is visibly shorter in comparison (Figure 1C and D) and, as a result, CptN_Er_ more closely resembles the structure of the Type II Toxin Kid.

**Figure 2.**
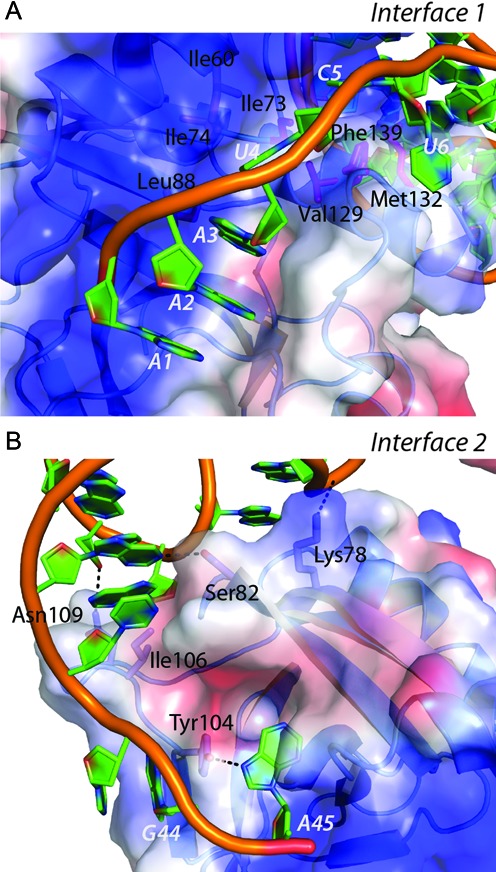
Diversity in the two protein–RNA interfaces in CptIN_Er_. (**A**) The surface representation shows the electrostatic potential (red: electronegative, blue: electropositive. Interface 1 is dominated by a long electropositive tract created by helix H3 interacting with the phosphate backbone, as well as the stabilization of U4 and C6 by a hydrophobic groove created through the labelled side chains. (**B**) Interface 2 lacks any dominant electropositive surface from the protein and is instead formed through a number of specific polar interactions with hydrophilic residues on the outside of the protein. Key residues are labelled and shown as sticks, with hydrogen bonds shown as black dashed lines. An important hydrophobic interaction is made to the proximal end of the RNA duplex by Ile106.

Each CptN_Er_ protein binds two CptI_Er_ RNAs, and the protein–RNA interfaces are considerable for both. Interface 1, which connects the protein to the 5′ end of the RNA, is the larger of the two and spans over 1250 Å^2^ (Figure 2A). Of note here is the strong electropositive tract (shown in blue) created by the RNA-facing side of helix H3, stabilizing the phosphate backbone and allowing the bases to stack and face into the protein. Also of note are the bases U4 and C6, which do not stack with adjacent bases, but are instead bound in hydrophobic pockets (residues Ile60, Ile74, Leu88 and Ile73, Val129, Phe139, Met132 respectively) that would otherwise be exposed to solvent. Interface 2, which spans an area of just over 1000 Å^2^, is made up of largely polar contacts between the protein and exposed bases at the 3′ end of CptI_Er_ (Figure 2B). A predicted hydrophobic interaction with Ile106 presumably helps to stabilize the proximal end of the S2 duplex, and the Tyr104 residue appears to be important both in helping to stabilize the position of the scissile A45 nucleotide, as well as stacking onto G44.

**Figure 3.**
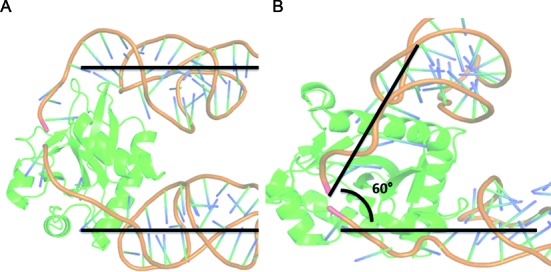
The heterotetrameric quaternary structure and the geometry of the bound RNA. (**A**) It can be seen that the incoming and outgoing RNA strands in CptIN_Er_ are completely parallel, driven by a much more extensive single stranded region at both the 5′ and 3′ ends of CptI_Er_ looping into the protein. (**B**) In ToxIN_Pa_, the interfaces are much more direct in relation to the ToxI_Pa_ duplex, creating a 60° angle between adjacent ToxI_Pa_ strands, leading to the formation of a heterohexamer.

The angle that the protein component makes with the antitoxin strands is related to the quaternary state as it forces the complex into a particular geometry; the angle that CptI_Er_ makes with CptN_Er_ is significantly smaller than that between ToxI and ToxN (Figure 3), and the number of amino acid residues that are involved in contacting the RNA ligand is high.

### The CptI_Er_ RNA is an H-type pseudoknot with a functionally important extended A-minor twist and no major groove loop

**Figure 4.**
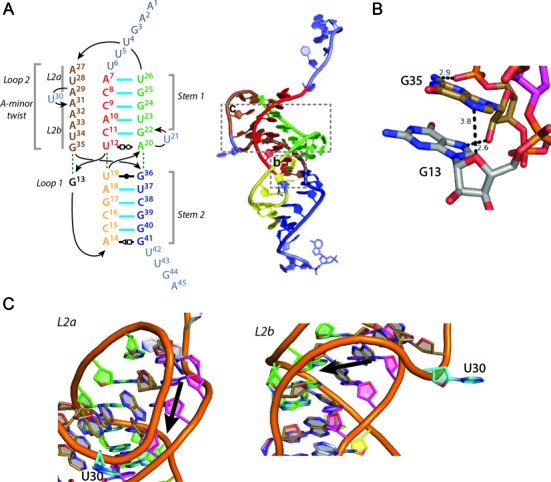
CptI_Er_ is an H-Type pseudoknot with unique features in its L1 and L2 loop regions. (**A**) Left: a coloured schematic of CptI_Er_ showing its two stems (S1 and S2), and two loop regions (L1 and L2); right: a representation of the schematic on a cartoon of a single CptI_Er_ monomer. (**B**) A close-up of the L1 loop region, unique in its dissociation with the stems altogether. It instead stacks with the proximal end of the L2 loop. (**C**) Close-up cartoon representations of the L2 loop region (brown), which can be divided into two halves—L2a and L2b. Above: the upper half (L2a) is a unique counterclockwise A-minor twist; below: the lower half (L2b) is a clockwise A-minor twist, with the change in directionality mediated by the ‘kink’ nucleotide (U30, teal).

The CptI_Er_ RNA observed bound to CptN_Er_ is a classic H-type pseudoknot of 45 nt, which is derived from the two genetic *cptI_Er_* repeats. The CptI_Er_ pseudoknot comprises two coaxial stem (base-paired) regions S1 and S2, with two loops L1 and L2, with L1 being extremely short (one base), and a much longer L2 loop (Figure 4A). The previously-solved Type III antitoxin structures—the ToxI RNAs from *Pectobacterium* and *Bacillus* (14,15)—were found to form very similar H-type pseudoknots, and the tertiary structures of both were maintained by intricate triplex interactions (which were largely conserved between the two antitoxins) between their loop sections and the associated stems. While the *Pectobacterium* and *Bacillus* ToxI structures are very similar to each other, the fold of the CptI_Er_ differs markedly from the ToxI paradigm, and is also structurally extremely unusual among known RNA pseudoknots notably in the structure of the loops.

**Figure 5.**
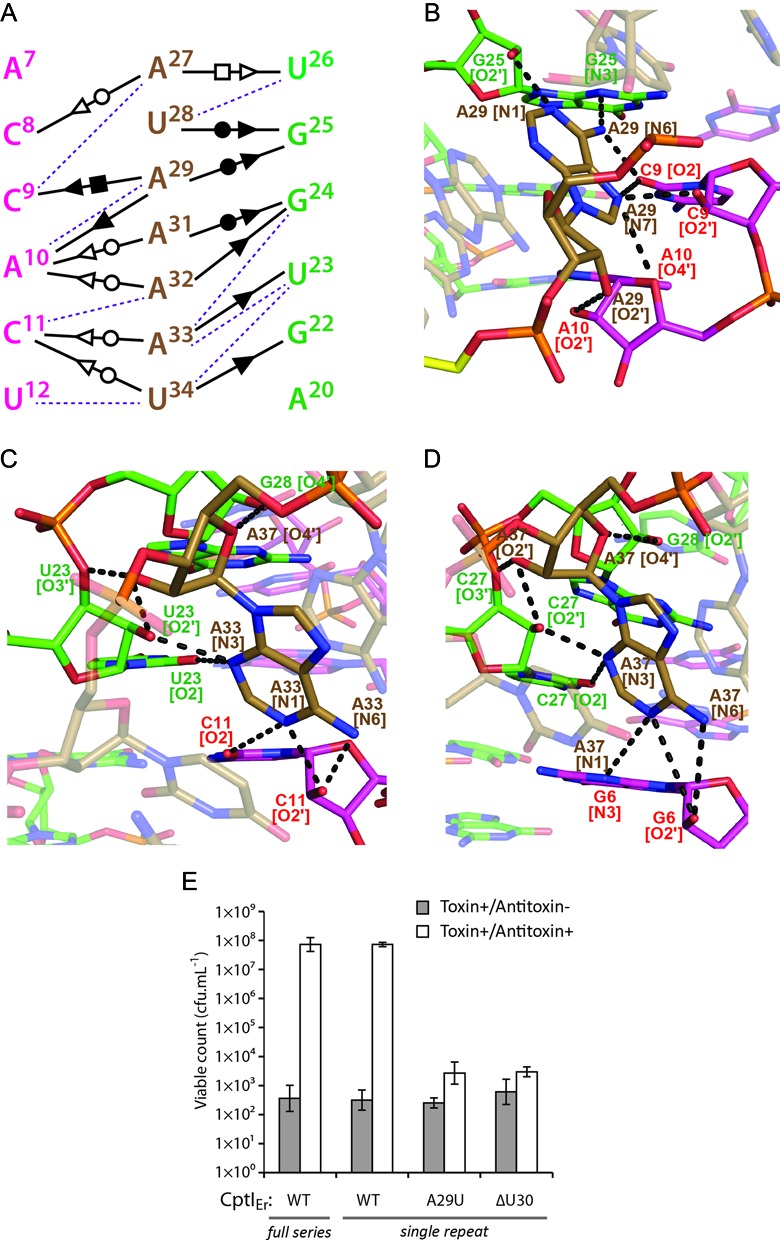
The A-minor twist motif is stabilized by extensive, multi-tiered, hydrogen bond networks and is required for antitoxicity. (**A**) A schematic of the complex and interactions observed in the A-minor twist motif seen in isolation from the rest of the pseudoknot. Purple dashes represent unusual polar interactions with no known notation, representing interactions involving the O3’ or O4’ of the ribose sugar. (**B**) Interactions in the counterclockwise A-minor twist are dominated by interactions with the backbone of the red half of the stem (A7–10). Black dashes represent hydrogen bonds. The U30 kink nucleotide is again highlighted here in yellow. (**C**) The opposite is seen in the clockwise A-minor twist, where the strongest networks are formed on the green half of the stem (G22–24). (**D**) An identical network can be seen in a previously identified A-minor motif in an unrelated structured RNA, the SAM-II riboswitch (PDB: 2QWY). (**E**) Growth of *Escherichia coli* DH5α strains overexpressing CptN_Er_ and CptI_Er_. Overexpression of CptN_Er_ prevents growth, which is rescued by co-overexpression of the full series of CptI_Er_ repeats. A single CptI_Er_ repeat, corresponding to the processed unit observed in the crystal structure, shows full antitoxicity. However, mutants CptI_Er_-A29U and CptI_Er_-ΔU30 are unable to counteract the toxicity of CptN_Er_. Results shown are mean ± standard deviation for three biological replicates.

In most pseudoknots the L1 loop interacts with the major groove of the S2 helix, and L2 interacts with the minor groove of the S1 helix. This topology often leads to the triplex interactions seen in many pseudoknots. In CptI_Er_, the single base that forms the L1 loop, G13, does not interact with the major groove of S2 at all and is held in place by base stacking with the 3′ end of the L2 loop (G35), at the same time forming some unique intra-chain hydrogen bonds (Figure 4B). The L2 loop (A27-G35) in CptI_Er_ is much longer than those of the ToxIs, and is very A-rich. Whilst L2 follows the classical association with the minor groove of S1, the details of the interaction are not standard (Figures 4C and 5). L2 forms a short, three-stranded helix which has been previously referred to as an A-minor twist motif (21). In other RNA species, the widely occurring A-minor motif involves interaction between an unpaired adenine base with the minor groove of an RNA duplex, usually involving interactions with both the exposed bases in the groove as well as the RNA backbone. A-minor twist motifs can be seen as extensions of the classical A-minor types and were first seen in the SAM-II riboswitch (36) and as part of the *glmS* ribozyme structure (37). Both structures reported a series of adenine bases associated with the minor groove of a duplex at an angle of ∼70° with respect to the duplex helical axis. The L2 loop in CptI_Er_ is similar, but, being far longer than either of the previously described A-minor twists, shows further detail as to how this motif can be extended. The motifs in the SAM-II riboswitch and in *glmS* were shown to rotate around the helix clockwise. Similarly, the section of the loop marked L2b (A31-G35) also rotates around S1 in a clockwise manner (Figure 4C), with the polar interactions surrounding one particular nucleotide, A33, and the similarity of these interactions to those surrounding A37 in the riboswitch structure reflecting this (Figure 5C and D). As well as the nature of the interactions being made with multiple ‘tiers’ of the S1 duplex, a number of interesting features of L2b should be noted, including the intricate hydrogen bond network centred on the 2′ hydroxyl oxygen atoms of A33 and U23, as well as an unusual interaction between the 4′ oxygen of A33 and the 2′ hydroxyl of G24 (Figure 5A and C).

In contrast to L2b, L2a (A27–A29) is a counter-clockwise twist of 3 nt with its own unique set of polar interactions (Figure 5A); this displays an equally extended network of hydrogen bonds as exemplified by the schematic shown for the nucleotide A29 (Figure 5B). Of note is the fact that this counter-clockwise twist was not seen adjacent to the A-minor twist in *glmS* or the SAM-II riboswitch. To determine whether this counter-clockwise twist had been seen in any previously published RNA structures, a search was performed using the calculated torsional angles (Supplementary Table S2) for nucleotides A27–A29. No examples of nucleotide stretches with similar torsional angles (total deviation <40) that also interacted with the minor groove of a double helix were found in any previously described RNA structure. This suggests that the counter-clockwise A-minor twist is a novel motif.

A schematic of all the interactions in the CptI A-minor twist summarizes the extensive network encompassing the entirety of L2 (Figure 5A). The only bases that do not participate in the network directly are U30, which faces outwards (and therefore makes no polar contacts with the pseudoknot itself) but is instrumental in changing the directionality of the twist, and G35, which appears to not make any interactions with the minor groove itself yet stacks with the rest of the bases in the twist, and acts to bridge the twist with the sole L1 nucleotide, G13 (Figure 4B). In both the reported examples of A-minor twist motifs (36,37), the segment was small. However, the potential for it to be lengthened further with the aid of a ‘kink’ nucleotide such as U30, as shown here, reveals the capacity for the A-minor twist to be extended into a dominant structural feature. To test the functional significance of the extended A-minor twist, site-directed mutants of CptI_Er_ were tested for antitoxicity against CptN_Er_ (Figure 5E). As shown, deletion of the ‘kink’ nucleotide U30, or substitution of the adjacent A29 for U, rendered CptI_Er_ unable to rescue the growth inhibition caused by CptN_Er_. This result suggests that the extended A-minor twist seen in the structure is important for CptI_Er_ function, and that the structural integrity of this feature depends on the presence of a ‘kink’ nucleotide to facilitate a change of direction of the twist, and on extensive hydrogen-bonding between A29 and the RNA duplex to stabilize the counter-clockwise portion of this motif.

### CptI RNAs show selective antitoxicity

**Figure 6.**
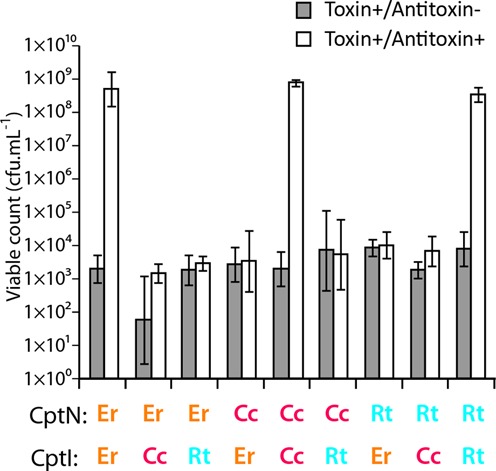
CptN toxins are specific for their cognate CptI antitoxins. Kill/rescue assay with different combinations of CptI and CptN. All three toxins could be neutralized by their cognate antitoxin, but not by the non-cognate CptIs. Results shown are mean ± standard deviation for three biological replicates.

The nature of the interactions observed at the protein–RNA interfaces of the CptIN complex suggested that antitoxin recognition within this family would be highly specific, as precise CptI–CptN contacts would be needed to maintain the inactive complex. To explore this hypothesis, cross-inhibition experiments between CptIN_Er_ components and the toxin and antitoxin components of the two other validated Type III TA systems of the CptIN family were performed. The three CptIN systems (CptIN_Er_; CptIN_Rt_ from *Ruminococcus torques* L2–14; and CptIN_Cc_ from *Coprococcus catus* GD-7) share a common locus organization and 35–44% sequence identity between their toxin components. Expression of any of the three CptN toxins was sufficient to cause a dramatic drop in viable count, and this was restored by co-overexpression of the cognate CptI antitoxin (Figure 6). Antitoxicity was not observed in any of the six strains expressing non-cognate CptI and CptN combinations. CptI RNA antitoxins are therefore highly selective toxin inhibitors. This exquisite selectivity was also observed in the ToxIN family (15) and supports the hypothesis that the enzyme and its substrate have co-evolved as a mutually compensating pair.

### The active site of CptN_Er_ is conserved with ToxN toxins

**Figure 7.**
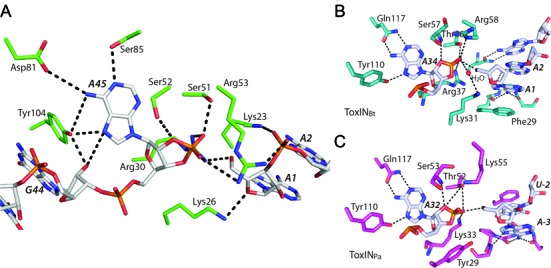
A conserved active site and mechanism for the RNase activity of CptN_Er_. (**A**) A stick representation of the active site residues surrounding the scissile adenine base (A45). Of note is Tyr104 which appears instrumental in both specifying for the adenine, as well as stacking onto the adjacent bases. (**B** and **C**) Show similar representations of the active sites from ToxN_Pa_ and ToxN_Bt_, showing a conserved mechanism and hydrogen bond cage, despite significant primary sequence differences elsewhere.

The RNA chains seen in the CptIN complex structure each correspond to single repeats from the repetitive CptI sequence and a cyclic phosphate is seen at the 3′ end of each RNA chain on the A45 nucleotide (Figure 7); these observations indicate that the CptN protein is, like ToxN, a ribonuclease that processes its own antitoxin. Although there is very little primary sequence identity between CptN_Er_ and any of the ToxNs, the hydrogen bonding networks in their RNase active sites are well conserved. However, some deviations can be seen. One is the use of two serine residues in the conserved triad of [Ser]-[Ser/Thr]-[Arg] instead of threonine as in the ToxNs. Another is in the group of residues specifying the adenine base at the site of cleavage: the coordinating glutamine is instead replaced by two residues (Asp81 and Ser85), and the coordinating tyrosine (Tyr104) approaches from below the plane of the base as depicted, instead of above, as in the ToxN structures. This positioning also stabilizes the entry of the RNA by stacking with G44, as described above. The combined evidence from the structures of, now, three active sites in complex with their RNA products suggest a catalytic mechanism that is similar to that seen in Ribonuclease A (38), with the residues mentioned conserved specifically to stabilize the generation of the cyclic phosphate. A similar mechanism was postulated in the resolution of the AbiQ structure (35).

### The TA locus CptIN stabilizes plasmids, but does not abort phage infection in *E. coli*

**Figure 8.**
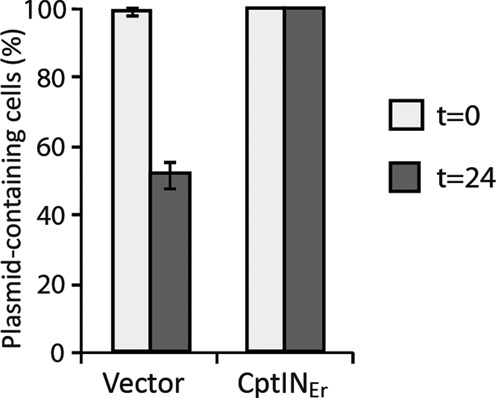
CptIN_Er_ stabilizes plasmids *in vivo*. Proportion of plasmid-containing *Escherichia coli* W3110 cells before (white) or after (grey) 24 h of non-selective exponential growth. Results shown are mean ± standard deviation for three biological replicates.

Type III TA systems of the ToxIN family have roles in bacteriophage resistance and plasmid stabilization (13,15,35). The *cptIN*_Er_ locus was therefore tested for capacity to confer these phenotypes on a bacterial host. In the absence of known *Eubacterium* phages, we assessed impacts on phage of the CptIN_Er_ system reconstituted in *E. coli*. To examine bacteriophage resistance, an *E. coli* strain expressing CptIN_Er_ was screened with over 200 environmentally isolated ‘virgin’ coliphages. However, no difference was seen in the plaque forming ability of these phages in comparison to the control strain without plasmid. To explore plasmid stabilization by *cptIN*_Er_, plasmid loss assays were performed in *E. coli* W3110 over the course of 24 h. As shown (Figure 8), the test vector was lost from ∼50% of the cells over this period, while the presence of *cptIN*_Er_ increased plasmid retention in the *E. coli* population to 100%. Although *cptIN*_Er_ is located on the chromosome in the original host, we suggest that its plasmid stabilization property is likely to also promote its retention in *E. rectale* and in other hosts that may receive this locus through horizontal transfer.

## DISCUSSION

The present work is the first structural study of a new family of Type III loci, the CptINs, originally identified in a bioinformatics search for the distribution of Type III TA systems. With the precedent set by the ToxIN system structures (the two ToxINs from *Pectobacterium* (14) and *Bacillus* (15), and the AbiQ structure from *Lactococcus* (35)) of the formation of a heterohexameric triangular oligomer, it was expected that the CptIN and TenpIN families would also exhibit this complex common architecture; indeed the other Type III families were found using the structure of ToxN_Pa_ as the initial seed in the bioinformatic search (16).

Unexpectedly, the CptIN_Er_ structure solved here forms a heterotetrameric complex (Figure 1B), which was validated by analytical size exclusion chromatography (Supplementary Figure S1). While the biological reason behind the differing organization of the tetrameric and hexameric Type III TA systems remains unknown, it is not unreasonable to suggest that their respective quaternary structures may play a role in their relative stabilities within the cell. A higher order complex necessitates a greater entropic penalty in its formation and thus may be more likely to be disrupted and dissociate in the cell.

The CptI_Er_ structure adds evidence to the now increasing perception that RNA molecules, like proteins, can form diverse structures to meet requirements for molecular recognition (17). The A-minor triple helix in the form of two adjacent A-minor twist motifs (Figures 4 and 5) is truly remarkable in the multiple levels of interactions that are formed in order to stabilize the duplex, and along with it, the pseudoknot itself. The occurrence of the A-minor twist in the SAM-II riboswitch lends strength to the idea that there are a limited number of RNA folds used in nature. With all Type III antitoxins to date found to have a pseudoknot structure, it could be that the features of pseudoknots are both sufficient and necessary for its functionality; the complex intramolecular interactions allow for the generation of a large interacting surface, while the single stranded nature of the 5′ and 3′ ends allow for processing by a co-factor. Nonetheless, taking into account the number of unusual interactions seen (hydrogen bonding to the 3′ and 4′ ribose oxygens), this CptI_Er_ structure illustrates the variety of intra-strand RNA interactions that can occur.

This particular structure also raises some interesting questions regarding the evolution of the Type III TA systems. The conservation of the cage of residues in the active site shows the importance of this core to the toxic function of the system, but the low primary sequence identity (<15% between CptN_Er_ to either of the ToxNs) overall implies a high degree of malleability when nucleating the secondary structure around it. The three Type III families seen appear to therefore represent three energetically favourable ‘solutions’ to forming this protein–RNA complex. The lack of cross-reactivity between even extremely similar antitoxin RNAs (Figure 6) show that anything that would disrupt the TA association while maintaining toxicity should ultimately lead to a lower fitness for the cell. It therefore seems that any deleterious mutation in one component must be compensated by mutation in the same or partner component. As one component is an enzyme and the other the target substrate, this system represents a distinctive case in molecular evolution in which substrates co-vary with the enzyme.

Functionally, there are still many mysteries surrounding TA systems. The plasmid stabilization shows the selfish nature of these loci, independent of host context. While no phage resistance was seen in the experiments conducted in *E. coli*, the fact that such structural diversity is seen, even with the integrity of the TA functionality being so critical, hints at the importance of these loci in bacterial physiology.

## Supplementary Material

SUPPLEMENTARY DATA
