# Supplementary material for: Co-evolution of quaternary organization and novel RNA tertiary interactions revealed in the crystal structure of a bacterial protein–RNA toxin–antitoxin system
Source: Nucleic Acids Res. 2015 Sep 8;43(19):9529–40. doi: 10.1093/nar/gkv868 (PMC4627078; doi:10.1093/nar/gkv868)
Supplement: SUPPLEMENTARY DATA [file supp_43_19_9529__index.html]

Co-evolution of quaternary organization and novel RNA tertiary interactions revealed in the crystal structure of a bacterial protein–RNA toxin–antitoxin system — Co-evolution of quaternary organization and novel RNA tertiary interactions revealed in the crystal structure of a bacterial protein–RNA toxin–antitoxin system — SUPPLEMENTARY DATA 

# Co-evolution of quaternary organization and novel RNA tertiary interactions revealed in the crystal structure of a bacterial protein–RNA toxin–antitoxin system

## SUPPLEMENTARY DATA

- SUPPLEMENTARY DATA
- SUPPLEMENTARY DATA
