# Supplementary material for: Co-evolution of quaternary organization and novel RNA tertiary interactions revealed in the crystal structure of a bacterial protein–RNA toxin–antitoxin system
Source: Nucleic Acids Res. 2015 Sep 8;43(19):9529–40. doi: 10.1093/nar/gkv868 (PMC4627078; doi:10.1093/nar/gkv868)
Supplement: SUPPLEMENTARY DATA [file supp_gkv868_nar-00543-z-2015-File010.pdf]

Supplementary Table 1 Oligomerization calculations from size exclusion chromatography data

**Complex**	**Mass from SEC (Da)**	**Calculated mass of Protein/RNA dimer (Da)**	**Number of Protein/RNA dimers in complex**
CptIN	77259	33931	2.28
ToxIN	110630	31367	3.53

Supplementary Table 2 Torsion angles of counter-clockwise twist in $CptI_{Er}$

**Residue**	**Eta (degree)**	**Theta (degree)**
Chain B A27	197.4	145.5
Chain B A28	145.3	179.5
Chain B A29	188.2	154.5

Supplementary Figure Legends

Supplementary Figure 1 - Interactions with symmetry cells show a heterotetrameric biological unit

One full asymmetric unit from the crystal structure is shown in cyan, with two symmetry units in light grey and dark grey. Two complete heterotetrameric units are shown opaque above the rest of the chains.

Supplementary Figure 2 - Analytical size exclusion chromatography of CptIN$_{Er}$

(A) The trace is shown for the elution of the CptIN$_{Er}$ complex through a Superdex S200 13/30 (GE Healthcare) column. CptIN$_{Er}$ elutes as a single peak. (B) Molecular weight standards were run to calculate a standard curve, for which the R2 value is shown. Values for the molecular weight of ToxIN$_{Pa}$ (known to be a heterohexamer) and CptIN$_{Er}$ were calculated from the points marked on the curve, and results displayed in Supplementary Table 1.

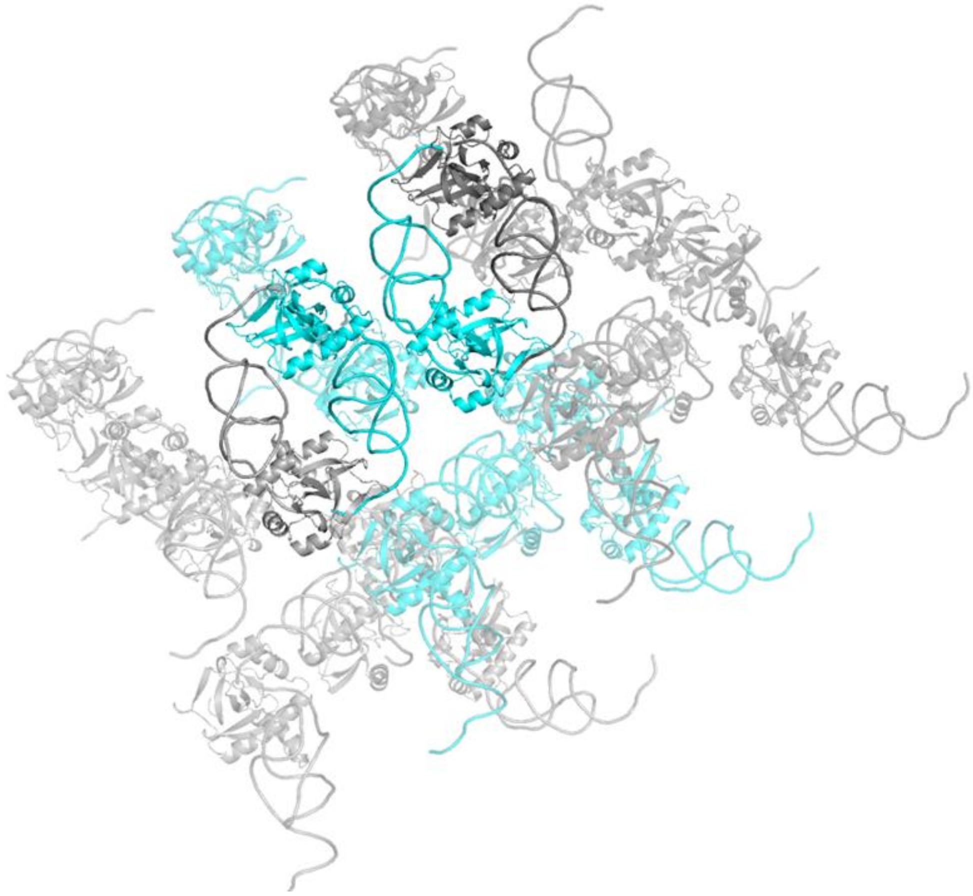

Supplementary Figure 1

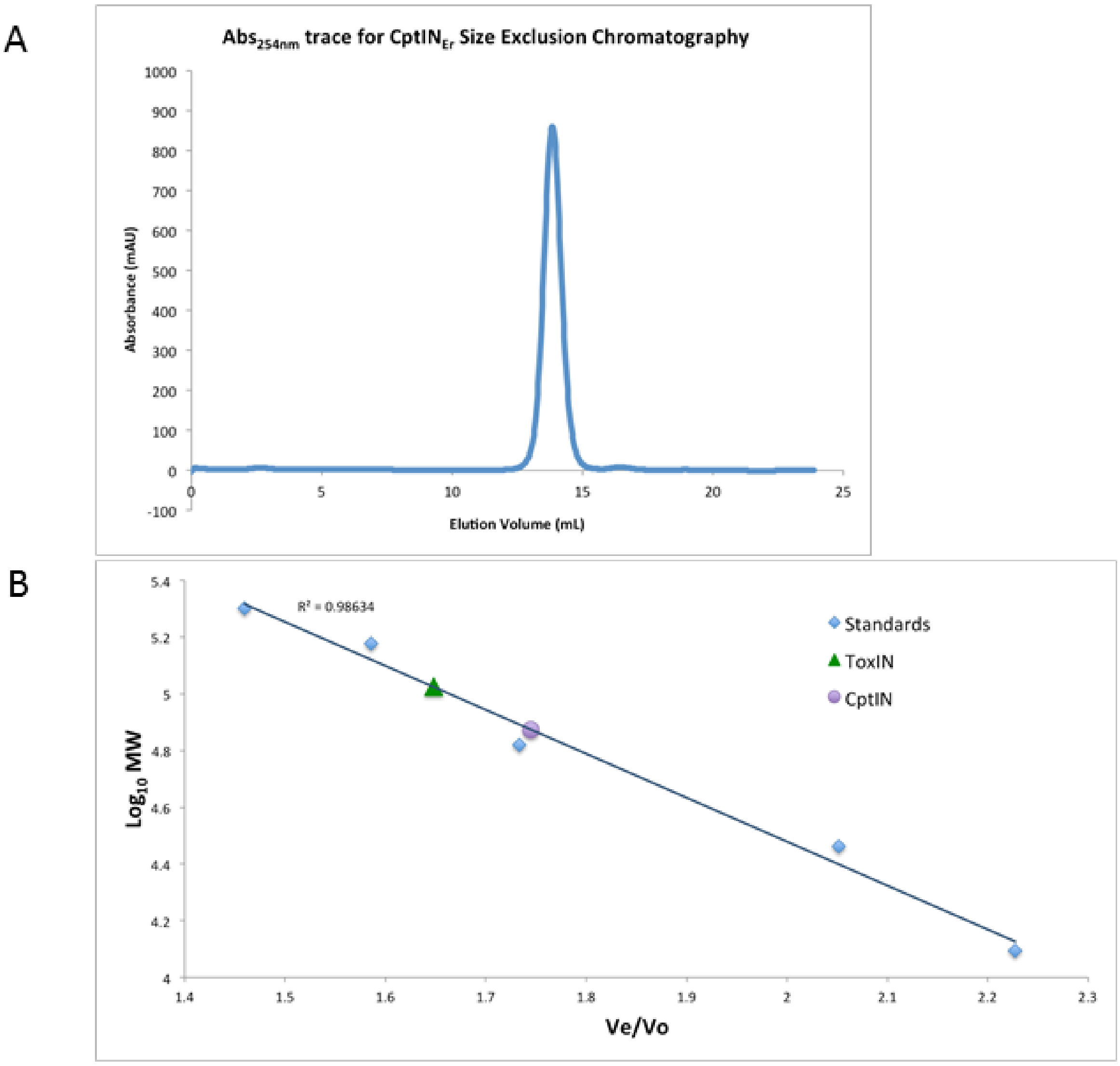

Supplementary Figure 2
