# Supplementary material for: Co-evolution of quaternary organization and novel RNA tertiary interactions revealed in the crystal structure of a bacterial protein–RNA toxin–antitoxin system
Source: Nucleic Acids Res. 2015 Sep 8;43(19):9529–40. doi: 10.1093/nar/gkv868 (PMC4627078; doi:10.1093/nar/gkv868)
Supplement: SUPPLEMENTARY DATA [file supp_gkv868_nar-00543-z-2015-File011.pdf]

wwPDB X-ray Structure Validation Summary Report (i)

Oct 30, 2014 – 02:57 PM EDT

PDB ID	:	4RMO
Title	:	Crystal Structure of the CptIN Type III Toxin-Antitoxin System from Eubacterium rectale
Authors	:	Rao, F.; Voss, J.E.; Short, F.L.; Luisi, B.F.
Deposited on	:	2014-10-21
Resolution	:	2.20 Å(reported)

DISCLAIMER

This is a preliminary version of the new style of wwPDB validation report.
We welcome your comments at validation@mail.wwpdb.org
A user guide is available at http://wwpdb.org/ValidationPDFNotes.html

CONFIDENTIAL VALIDATION REPORT

The following versions of software and data (see references) were used in the production of this report:

MolProbity	:	4.02b-467
Mogul	:	1.16 November 2013
Xtriage (Phenix)	:	dev-1439
EDS	:	stable24055
Percentile statistics	:	21963
Refmac	:	5.8.0049
CCP4	:	6.1.3
Ideal geometry (proteins)	:	Engh & Huber (2001)
Ideal geometry (DNA, RNA)	:	Parkinson et. al. (1996)
Validation Pipeline (wwPDB-VP)	:	stable24055

1 Overall quality at a glance (i)

The reported resolution of this entry is 2.20 Å.

Percentile scores (ranging between 0-100) for global validation metrics of the entry are shown in the following graphic. The table shows the number of entries on which the scores are based.

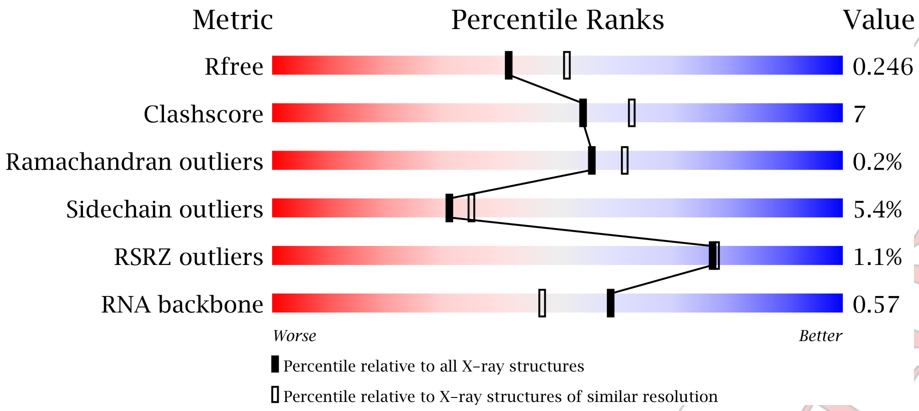

Metric	Whole archive (#Entries)	Similar resolution (#Entries, resolution range(Å))
R_{free}	66092	2938 (2.20-2.20)
Clashscore	79885	3751 (2.20-2.20)
Ramachandran outliers	78287	3681 (2.20-2.20)
Sidechain outliers	78261	3682 (2.20-2.20)
RSRZ outliers	66119	2939 (2.20-2.20)
RNA backbone	1838	1120 (3.00-1.40)

The table below summarises the geometric issues observed across the polymeric chains and their fit to the electron density. The red, orange, yellow and green segments on the lower bar indicate the fraction of residues that contain outliers for >=3, 2, 1 and 0 types of geometric quality criteria. The upper red bar (where present) indicates the fraction of residues that have poor fit to the electron density.

Mol	Chain	Length	Quality of chain
1	A	155	
1	C	155	
1	E	155	
1	G	155	
1	I	155	
1	K	155	
1	M	155	
1	O	155	
2	B	45	
2	D	45	
2	F	45	

Continued on next page...

CONFIDENTIAL VALIDATION REPORT

Continued from previous page...

Mol	Chain	Length	Quality of chain
2	H	45	
2	J	45	
2	L	45	
2	N	45	
2	P	45	

The following table lists non-polymeric compounds that are outliers for geometric or electron-density-fit criteria:

Mol	Type	Chain	Res	Geometry	Electron density
3	CA	D	104	-	X
3	CA	J	104	-	X
3	CA	N	104	-	X

CONFIDENTIAL VALIDATION REPORT

2 Entry composition (i)

There are 4 unique types of molecules in this entry. The entry contains 19478 atoms, of which 0 are hydrogen and 0 are deuterium.

In the tables below, the ZeroOcc column contains the number of atoms modelled with zero occupancy, the AltConf column contains the number of residues with at least one atom in alternate conformation and the Trace column contains the number of residues modelled with at most 2 atoms.

- Molecule 1 is a protein called CptN Toxin.

Mol	Chain	Residues	Atoms						ZeroOcc	AltConf	Trace
			Total	C	N	O	S	Se			
1	A	155	1303	832	230	234	2	5	0	1	0
1	C	155	1303	832	230	234	2	5	0	1	0
1	E	155	1303	832	230	234	2	5	0	1	0
1	G	155	1303	832	230	234	2	5	0	1	0
1	I	155	1293	827	226	233	2	5	0	1	0
1	K	155	1303	832	230	234	2	5	0	1	0
1	M	155	1300	830	229	234	2	5	0	1	0
1	O	155	1303	832	230	234	2	5	0	1	0

- Molecule 2 is a RNA chain called RNA (45-MER).

Mol	Chain	Residues	Atoms					ZeroOcc	AltConf	Trace
			Total	C	N	O	P			
2	B	45	962	430	171	316	45	0	0	0
2	D	45	962	430	171	316	45	0	0	0
2	F	45	962	430	171	316	45	0	0	0
2	H	45	962	430	171	316	45	0	0	0
2	J	45	962	430	171	316	45	0	0	0
2	L	45	962	430	171	316	45	0	0	0

Continued on next page...

CONFIDENTIAL VALIDATION REPORT

Continued from previous page...

Mol	Chain	Residues	Atoms					ZeroOcc	AltConf	Trace
2	N	45	Total 962	C 430	N 171	O 316	P 45	0	0	0
2	P	45	Total 962	C 430	N 171	O 316	P 45	0	0	0

- Molecule 3 is CALCIUM ION (three-letter code: CA) (formula: Ca).

Mol	Chain	Residues	Atoms		ZeroOcc	AltConf
3	P	4	Total 4	Ca 4	0	0
3	G	1	Total 1	Ca 1	0	0
3	J	6	Total 6	Ca 6	0	0
3	D	7	Total 7	Ca 7	0	0
3	K	1	Total 1	Ca 1	0	0
3	H	7	Total 7	Ca 7	0	0
3	B	6	Total 6	Ca 6	0	0
3	N	6	Total 6	Ca 6	0	0
3	L	6	Total 6	Ca 6	0	0
3	F	5	Total 5	Ca 5	0	0

- Molecule 4 is water.

Mol	Chain	Residues	Atoms		ZeroOcc	AltConf
4	A	82	Total 82	O 82	0	0
4	B	99	Total 99	O 99	0	0
4	C	88	Total 88	O 88	0	0
4	D	97	Total 97	O 97	0	0
4	E	53	Total 53	O 53	0	0

Continued on next page...

CONFIDENTIAL VALIDATION REPORT

Continued from previous page...

Mol	Chain	Residues	Atoms	ZeroOcc	AltConf
4	F	67	Total O<br>67 67	0	0
4	G	113	Total O<br>113 113	0	0
4	H	100	Total O<br>100 100	0	0
4	I	82	Total O<br>82 82	0	0
4	J	100	Total O<br>100 100	0	0
4	K	89	Total O<br>89 89	0	0
4	L	85	Total O<br>85 85	0	0
4	M	54	Total O<br>54 54	0	0
4	N	82	Total O<br>82 82	0	0
4	O	68	Total O<br>68 68	0	0
4	P	63	Total O<br>63 63	0	0

CONFIDENTIAL VALIDATION REPORT

3 Residue-property plots ⓘ

These plots are drawn for all protein, RNA and DNA chains in the entry. The first graphic for a chain summarises the proportions of errors displayed in the second graphic. The second graphic shows the sequence view annotated by issues in geometry and electron density. Residues are color-coded according to the number of geometric quality criteria for which they contain at least one outlier: green = 0, yellow = 1, orange = 2 and red = 3 or more. A red dot above a residue indicates a poor fit to the electron density (RSRZ > 2). Stretches of 2 or more consecutive residues without any outlier are shown as a green connector. Residues present in the sample, but not in the model, are shown in grey.

• Molecule 1: CptN Toxin

Chain A:

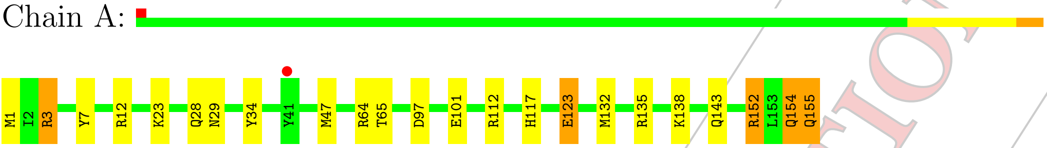

• Molecule 1: CptN Toxin

Chain C:

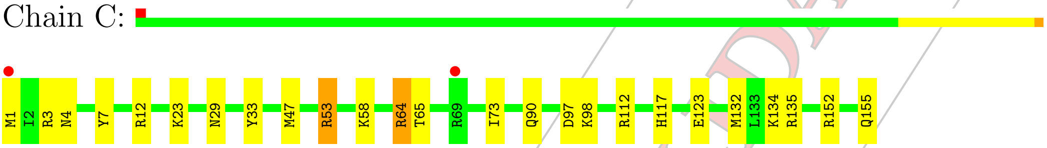

• Molecule 1: CptN Toxin

Chain E:

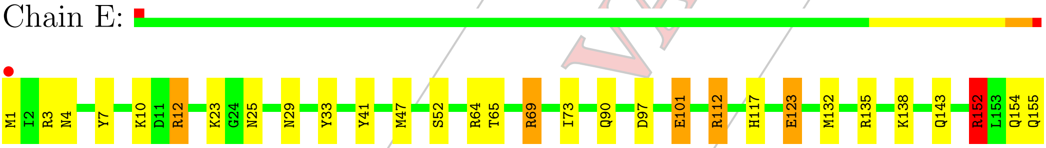

• Molecule 1: CptN Toxin

Chain G:

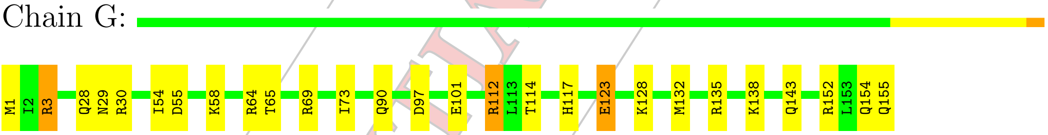

• Molecule 1: CptN Toxin

Chain I:

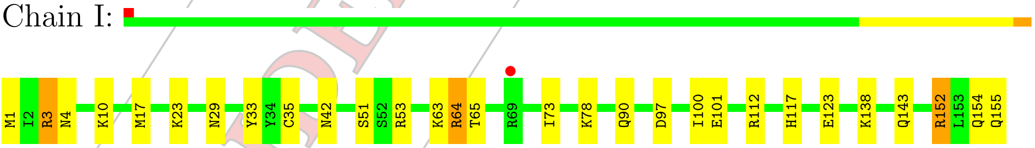

• Molecule 1: CptN Toxin

Chain K:

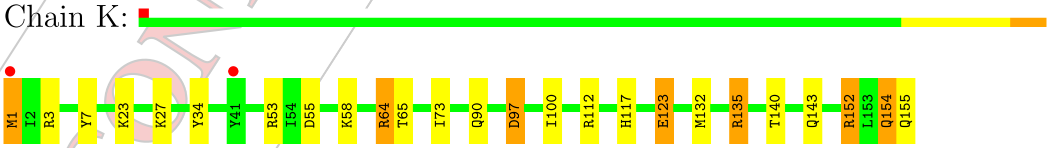

CONFIDENTIAL VALIDATION REPORT

- Molecule 1: CptN Toxin

Chain M:

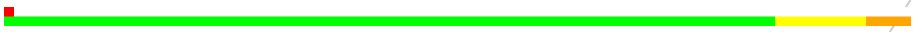

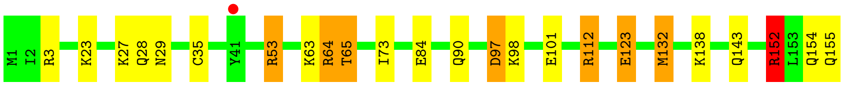

- Molecule 1: CptN Toxin

Chain O:

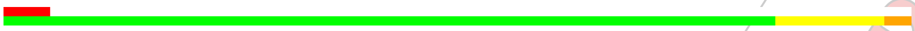

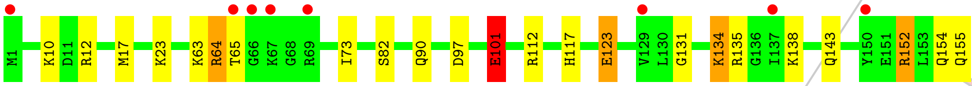

- Molecule 2: RNA (45-MER)

Chain B:

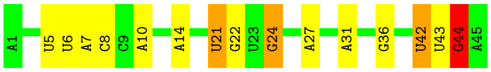

- Molecule 2: RNA (45-MER)

Chain D:

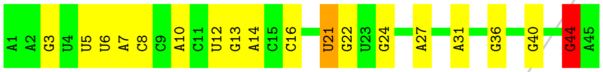

- Molecule 2: RNA (45-MER)

Chain F:

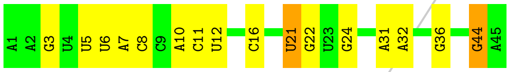

- Molecule 2: RNA (45-MER)

Chain H:

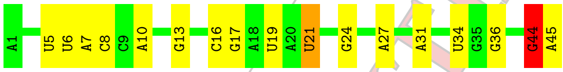

- Molecule 2: RNA (45-MER)

Chain J:

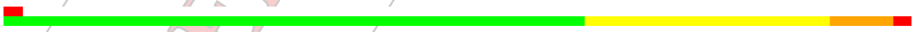

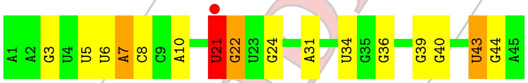

- Molecule 2: RNA (45-MER)

Chain L:

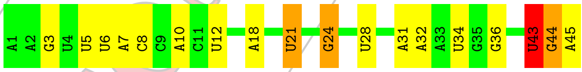

CONFIDENTIAL VALIDATION REPORT

• Molecule 2: RNA (45-MER)

Chain N:

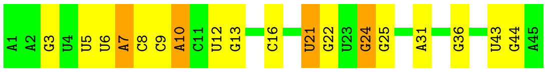

• Molecule 2: RNA (45-MER)

Chain P:

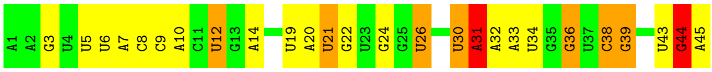

CONFIDENTIAL VALIDATION REPORT

4 Data and refinement statistics ⓘ

Property	Value	Source
Space group	P 1 21 1	Depositor
Cell constants<br>a, b, c, α, β, γ	63.11Å 185.89Å 138.78Å<br>90.00° 92.63° 90.00°	Depositor
Resolution (Å)	63.12 – 2.20<br>63.04 – 2.20	Depositor<br>EDS
% Data completeness<br>(in resolution range)	97.8 (63.12-2.20)<br>97.8 (63.04-2.20)	Depositor<br>EDS
R_{merge}	0.05	Depositor
R_{sym}	(Not available)	Depositor
$< I/\sigma(I) >$ [1]	2.51 (at 2.20Å)	Xtriage
Refinement program	REFMAC 5.7.0029	Depositor
R, R_{free}	0.199 , 0.234<br>0.212 , 0.246	Depositor<br>DCC
R_{free} test set	7919 reflections (5.28%)	DCC
Wilson B-factor (Å^2)	38.0	Xtriage
Anisotropy	0.052	Xtriage
Bulk solvent k_{sol}(e/Å^3), B_{sol}(Å^2)	0.35 , 31.7	EDS
Estimated twinning fraction	0.042 for h,-k,-l	Xtriage
L-test for twinning	$< \|L\| > = 0.49$, $< L^2 > = 0.32$	Xtriage
Outliers	0 of 157921 reflections	Xtriage
F_o,F_c correlation	0.94	EDS
Total number of atoms	19478	wwPDB-VP
Average B, all atoms (Å^2)	41.0	wwPDB-VP

Xtriage's analysis on translational NCS is as follows: *The largest off-origin peak in the Patterson function is 3.45% of the height of the origin peak. No significant pseudotranslation is detected.*

[1] Intensities estimated from amplitudes.

CONFIDENTIAL VALIDATION REPORT

5 Model quality ⓘ

5.1 Standard geometry ⓘ

Bond lengths and bond angles in the following residue types are not validated in this section: CA, A23

The Z score for a bond length (or angle) is the number of standard deviations the observed value is removed from the expected value. A bond length (or angle) with $|Z| > 5$ is considered an outlier worth inspection. RMSZ is the root-mean-square of all Z scores of the bond lengths (or angles).

Mol	Chain	Bond lengths		Bond angles	
		RMSZ	#$\|Z\|$ >5	RMSZ	#$\|Z\|$ >5
1	A	0.84	4/1331 (0.3%)	0.92	4/1776 (0.2%)
1	C	0.86	0/1331	1.01	12/1776 (0.7%)
1	E	0.81	2/1331 (0.2%)	0.94	7/1776 (0.4%)
1	G	0.90	5/1331 (0.4%)	0.95	5/1776 (0.3%)
1	I	0.85	0/1321	0.91	3/1764 (0.2%)
1	K	0.90	5/1331 (0.4%)	1.03	8/1776 (0.5%)
1	M	0.87	6/1328 (0.5%)	0.94	7/1773 (0.4%)
1	O	0.80	2/1331 (0.2%)	0.90	7/1776 (0.4%)
2	B	0.67	1/1048 (0.1%)	0.98	3/1632 (0.2%)
2	D	0.60	0/1048	0.92	4/1632 (0.2%)
2	F	0.54	0/1048	0.91	4/1632 (0.2%)
2	H	0.51	0/1048	0.90	3/1632 (0.2%)
2	J	0.72	1/1048 (0.1%)	0.98	6/1632 (0.4%)
2	L	0.58	0/1048	0.92	5/1632 (0.3%)
2	N	0.64	0/1048	0.98	6/1632 (0.4%)
2	P	0.76	3/1048 (0.3%)	1.33	18/1632 (1.1%)
All	All	0.76	29/19019 (0.2%)	0.97	102/27249 (0.4%)

Chiral center outliers are detected by calculating the chiral volume of a chiral center and verifying if the center is modelled as a planar moiety or with the opposite hand. A planarity outlier is detected by checking planarity of atoms in a peptide group, atoms in a mainchain group or atoms of a sidechain that are expected to be planar.

Mol	Chain	#Chirality outliers	#Planarity outliers
1	A	0	1
1	K	0	1
1	O	0	1
2	B	0	1
2	D	0	1
2	H	0	1
2	P	0	1

Continued on next page...

CONFIDENTIAL VALIDATION REPORT

Continued from previous page...

Mol	Chain	#Chirality outliers	#Planarity outliers
All	All	0	7

The worst 5 of 29 bond length outliers are listed below:

Mol	Chain	Res	Type	Atoms	Z	Observed(Å)	Ideal(Å)
2	B	42	U	O3'-P	-10.66	1.48	1.61
2	P	19	U	O3'-P	8.94	1.71	1.61
2	P	44	G	O5'-C5'	-7.43	1.30	1.42
2	J	40	G	O5'-C5'	-6.01	1.33	1.42
1	M	123[A]	GLU	CD-OE1	5.83	1.32	1.25

The worst 5 of 102 bond angle outliers are listed below:

Mol	Chain	Res	Type	Atoms	Z	Observed(^{o})	Ideal(^{o})
2	P	44	G	O5'-P-OP1	-16.77	90.58	110.70
2	B	42	U	C2'-C3'-O3'	14.73	141.91	109.50
2	P	44	G	O5'-P-OP2	11.68	124.71	110.70
2	P	44	G	C5'-C4'-O4'	-11.51	95.29	109.10
2	P	38	C	C2'-C3'-O3'	11.06	133.83	109.50

There are no chirality outliers.

5 of 7 planarity outliers are listed below:

Mol	Chain	Res	Type	Group
1	A	154	GLN	Peptide
2	B	44	G	Sidechain
2	D	44	G	Sidechain
2	H	44	G	Sidechain
1	K	154	GLN	Peptide

5.2 Close contacts (i)

In the following table, the Non-H and H(model) columns list the number of non-hydrogen atoms and hydrogen atoms in the chain respectively. The H(added) column lists the number of hydrogens added by MolProbity. The Clashes column lists the number of clashes within the asymmetric unit, and the number in parentheses is this value normalized per 1000 atoms of the molecule in the chain. The Symm-Clashes column gives symmetry related clashes, in the same way as for the Clashes column.

Mol	Chain	Non-H	H(model)	H(added)	Clashes	Symm-Clashes
1	A	1303	0	1315	23	0

Continued on next page...

CONFIDENTIAL VALIDATION REPORT

Continued from previous page...

Mol	Chain	Non-H	H(model)	H(added)	Clashes	Symm-Clashes
1	C	1303	0	1315	18	0
1	E	1303	0	1315	22	0
1	G	1303	0	1315	17	0
1	I	1293	0	1289	22	0
1	K	1303	0	1315	19	0
1	M	1300	0	1306	28	0
1	O	1303	0	1315	12	0
2	B	962	0	481	11	0
2	D	962	0	481	13	0
2	F	962	0	481	13	0
2	H	962	0	481	12	0
2	J	962	0	481	12	0
2	L	962	0	481	16	0
2	N	962	0	481	14	0
2	P	962	0	481	20	0
3	B	6	0	0	0	0
3	D	7	0	0	0	0
3	F	5	0	0	0	0
3	G	1	0	0	0	0
3	H	7	0	0	0	0
3	J	6	0	0	0	0
3	K	1	0	0	0	0
3	L	6	0	0	0	0
3	N	6	0	0	1	0
3	P	4	0	0	0	0
4	A	82	0	0	3	1
4	B	99	0	0	2	0
4	C	88	0	0	2	1
4	D	97	0	0	1	0
4	E	53	0	0	1	0
4	F	67	0	0	4	0
4	G	113	0	0	8	0
4	H	100	0	0	4	0
4	I	82	0	0	4	0
4	J	100	0	0	5	0
4	K	89	0	0	4	0
4	L	85	0	0	4	0
4	M	54	0	0	12	0
4	N	82	0	0	9	0
4	O	68	0	0	1	0
4	P	63	0	0	4	0
All	All	19478	0	14333	221	1

CONFIDENTIAL VALIDATION REPORT

Clashscore is defined as the number of clashes calculated for the entry per 1000 atoms (including hydrogens) of the entry. The overall clashscore for this entry is 7.

The worst 5 of 221 close contacts within the same asymmetric unit are listed below.

Atom-1	Atom-2	Distance(Å)	Clash(Å)
2:L:18:A:N7	4:L:212:HOH:O	1.58	1.32
1:M:53:ARG:CD	4:M:249:HOH:O	1.77	1.28
1:M:53:ARG:NE	4:M:249:HOH:O	1.67	1.21
2:N:10:A:OP2	4:N:261:HOH:O	1.66	1.13
1:A:1:MSE:HE3	1:A:7:TYR:CD2	1.93	1.04

All (1) symmetry-related close contacts are listed below. The label for Atom-2 includes the symmetry operator and encoded unit-cell translations to be applied.

Atom-1	Atom-2	Distance(Å)	Clash(Å)
4:A:226:HOH:O	4:C:277:HOH:O[1_455]	2.13	0.07

5.3 Torsion angles

5.3.1 Protein backbone (i)

In the following table, the Percentiles column shows the percent Ramachandran outliers of the chain as a percentile score with respect to all X-ray entries followed by that with respect to entries of similar resolution.

The Analysed column shows the number of residues for which the backbone conformation was analysed, and the total number of residues.

Mol	Chain	Analysed	Favoured	Allowed	Outliers	Percentiles	
1	A	154/155 (99%)	148 (96%)	5 (3%)	1 (1%)	33	32
1	C	154/155 (99%)	150 (97%)	4 (3%)	0	100	100
1	E	154/155 (99%)	149 (97%)	5 (3%)	0	100	100
1	G	154/155 (99%)	149 (97%)	5 (3%)	0	100	100
1	I	154/155 (99%)	151 (98%)	3 (2%)	0	100	100
1	K	154/155 (99%)	149 (97%)	4 (3%)	1 (1%)	33	32
1	M	154/155 (99%)	150 (97%)	4 (3%)	0	100	100
1	O	154/155 (99%)	149 (97%)	5 (3%)	0	100	100
All	All	1232/1240 (99%)	1195 (97%)	35 (3%)	2 (0%)	56	62

All (2) Ramachandran outliers are listed below:

CONFIDENTIAL VALIDATION REPORT

Mol	Chain	Res	Type
1	A	154	GLN
1	K	154	GLN

5.3.2 Protein sidechains ⓘ

In the following table, the Percentiles column shows the percent sidechain outliers of the chain as a percentile score with respect to all X-ray entries followed by that with respect to entries of similar resolution. The Analysed column shows the number of residues for which the sidechain conformation was analysed, and the total number of residues.

Mol	Chain	Analysed	Rotameric	Outliers	Percentiles	
1	A	143/137 (104%)	135 (94%)	8 (6%)	30	33
1	C	143/137 (104%)	137 (96%)	6 (4%)	40	48
1	E	143/137 (104%)	132 (92%)	11 (8%)	18	18
1	G	143/137 (104%)	137 (96%)	6 (4%)	40	48
1	I	139/137 (102%)	131 (94%)	8 (6%)	28	31
1	K	143/137 (104%)	132 (92%)	11 (8%)	18	18
1	M	142/137 (104%)	134 (94%)	8 (6%)	30	33
1	O	143/137 (104%)	132 (92%)	11 (8%)	18	18
All	All	1139/1096 (104%)	1070 (94%)	69 (6%)	31	28

5 of 69 residues with a non-rotameric sidechain are listed below:

Mol	Chain	Res	Type
1	I	3	ARG
1	I	152	ARG
1	O	101	GLU
1	I	65	THR
1	I	112	ARG

Some sidechains can be flipped to improve hydrogen bonding and reduce clashes. 5 of 20 such sidechains are listed below:

Mol	Chain	Res	Type
1	G	117	HIS
1	I	25	ASN
1	K	155	GLN
1	G	25	ASN
1	G	29	ASN

CONFIDENTIAL VALIDATION REPORT

5.3.3 RNA (i)

Mol	Chain	Analysed	Backbone Outliers	Pucker Outliers
2	B	44/45 (97%)	6 (13%)	2 (4%)
2	D	44/45 (97%)	5 (11%)	1 (2%)
2	F	44/45 (97%)	4 (9%)	1 (2%)
2	H	44/45 (97%)	4 (9%)	1 (2%)
2	J	44/45 (97%)	6 (13%)	1 (2%)
2	L	44/45 (97%)	5 (11%)	1 (2%)
2	N	44/45 (97%)	5 (11%)	0
2	P	44/45 (97%)	10 (22%)	1 (2%)
All	All	352/360 (97%)	45 (12%)	8 (2%)

5 of 45 RNA backbone outliers are listed below:

Mol	Chain	Res	Type
2	B	5	U
2	B	10	A
2	B	21	U
2	B	22	G
2	B	31	A

5 of 8 RNA pucker outliers are listed below:

Mol	Chain	Res	Type
2	F	21	U
2	P	38	C
2	J	21	U
2	D	21	U
2	H	21	U

5.4 Non-standard residues in protein, DNA, RNA chains (i)

8 non-standard protein/DNA/RNA residues are modelled in this entry.

In the following table, the Counts columns list the number of bonds (or angles) for which Mogul statistics could be retrieved, the number of bonds (or angles) that are observed in the model and the number of bonds (or angles) that are defined in the chemical component dictionary. The Link column lists molecule types, if any, to which the group is linked. The Z score for a bond length (or angle) is the number of standard deviations the observed value is removed from the expected value. A bond length (or angle) with $|Z| > 2$ is considered an outlier worth inspection. RMSZ is the root-mean-square of all Z scores of the bond lengths (or angles).

CONFIDENTIAL VALIDATION REPORT

Mol	Type	Chain	Res	Link	Bond lengths			Bond angles		
					Counts	RMSZ	#|Z| > 2	Counts	RMSZ	#|Z| > 2
2	A23	B	45	2	26,28,29	1.97	8 (30%)	40,43,46	2.13	12 (30%)
2	A23	D	45	2	26,28,29	1.61	5 (19%)	40,43,46	2.15	10 (25%)
2	A23	F	45	2	26,28,29	1.44	3 (11%)	40,43,46	2.04	10 (25%)
2	A23	H	45	2	26,28,29	1.59	6 (23%)	40,43,46	2.17	9 (22%)
2	A23	J	45	2	26,28,29	1.37	4 (15%)	40,43,46	2.07	10 (25%)
2	A23	L	45	2	26,28,29	2.92	9 (34%)	40,43,46	3.32	20 (50%)
2	A23	N	45	2	26,28,29	1.67	3 (11%)	40,43,46	2.28	11 (27%)
2	A23	P	45	2	26,28,29	1.62	5 (19%)	40,43,46	2.15	10 (25%)

In the following table, the Chirals column lists the number of chiral outliers, the number of chiral centers analysed, the number of these observed in the model and the number defined in the chemical component dictionary. Similar counts are reported in the Torsion and Rings columns. '-' means no outliers of that kind were identified.

Mol	Type	Chain	Res	Link	Chirals	Torsions	Rings
2	A23	B	45	2	-	0/8/35/36	0/4/4/4
2	A23	D	45	2	-	0/8/35/36	0/4/4/4
2	A23	F	45	2	-	0/8/35/36	0/4/4/4
2	A23	H	45	2	-	0/8/35/36	0/4/4/4
2	A23	J	45	2	-	0/8/35/36	0/4/4/4
2	A23	L	45	2	-	0/8/35/36	0/4/4/4
2	A23	N	45	2	-	0/8/35/36	0/4/4/4
2	A23	P	45	2	-	0/8/35/36	0/4/4/4

The worst 5 of 43 bond length outliers are listed below:

Mol	Chain	Res	Type	Atoms	Z	Observed(Å)	Ideal(Å)
2	L	45	A23	C4-N9	-11.25	1.21	1.37
2	P	45	A23	PC-O3'	4.84	1.63	1.58
2	N	45	A23	PC-O2'	4.45	1.63	1.58
2	L	45	A23	PC-O3'	4.37	1.62	1.58
2	H	45	A23	PC-O3'	4.23	1.62	1.58

The worst 5 of 92 bond angle outliers are listed below:

Mol	Chain	Res	Type	Atoms	Z	Observed(^{o})	Ideal(^{o})
2	L	45	A23	O4'-C1'-N9	9.84	129.51	108.10
2	L	45	A23	C5-C4-N3	9.58	135.34	125.98
2	D	45	A23	C5-C4-N3	-8.08	118.10	125.98
2	J	45	A23	C5-C4-N3	-7.24	118.92	125.98

Continued on next page...

CONFIDENTIAL VALIDATION REPORT

Continued from previous page...

Mol	Chain	Res	Type	Atoms	Z	Observed(^{o})	Ideal(^{o})
2	F	45	A23	C5-C4-N3	-7.22	118.94	125.98

There are no chirality outliers.

There are no torsion outliers.

There are no ring outliers.

5.5 Carbohydrates (i)

There are no carbohydrates in this entry.

5.6 Ligand geometry (i)

Of 49 ligands modelled in this entry, 49 are monoatomic - leaving 0 for Mogul analysis.

There are no bond length outliers.

There are no bond angle outliers.

There are no chirality outliers.

There are no torsion outliers.

There are no ring outliers.

5.7 Other polymers (i)

There are no such residues in this entry.

5.8 Polymer linkage issues

There are no chain breaks in this entry.

CONFIDENTIAL VALIDATION REPORT

6 Fit of model and data

6.1 Protein, DNA and RNA chains

In the following table, the column labelled '#RSRZ> 2' contains the number (and percentage) of RSRZ outliers, followed by percent RSRZ outliers for the chain as percentile scores relative to all X-ray entries and entries of similar resolution. The OWAB column contains the minimum, median, 95th percentile and maximum values of the occupancy-weighted average B-factor per residue. The column labelled 'Q< 0.9' lists the number of (and percentage) of residues with an average occupancy less than 0.9.

Mol	Chain	Analysed	<RSRZ>	#RSRZ>2			OWAB(Å^2)	Q<0.9
1	A	155/155 (100%)	-0.35	1 (0%)	86	88	21, 33, 62, 74	0
1	C	155/155 (100%)	-0.21	2 (1%)	74	74	21, 34, 66, 89	0
1	E	155/155 (100%)	-0.03	1 (0%)	86	88	27, 47, 77, 94	0
1	G	155/155 (100%)	-0.20	0	100	100	18, 30, 57, 76	0
1	I	155/155 (100%)	-0.12	1 (0%)	86	88	20, 35, 67, 104	0
1	K	155/155 (100%)	-0.21	2 (1%)	74	74	21, 32, 61, 67	0
1	M	155/155 (100%)	-0.06	1 (0%)	86	88	24, 40, 70, 90	0
1	O	155/155 (100%)	0.30	8 (5%)	26	26	25, 48, 97, 134	0
2	B	45/45 (100%)	-0.66	0	100	100	26, 32, 49, 86	0
2	D	45/45 (100%)	-0.47	0	100	100	22, 33, 49, 89	0
2	F	45/45 (100%)	-0.52	0	100	100	24, 39, 63, 98	0
2	H	45/45 (100%)	-0.52	0	100	100	25, 40, 60, 93	0
2	J	45/45 (100%)	-0.44	1 (2%)	59	59	23, 34, 48, 97	0
2	L	45/45 (100%)	-0.38	0	100	100	28, 38, 75, 102	0
2	N	45/45 (100%)	-0.35	0	100	100	24, 38, 65, 96	0
2	P	45/45 (100%)	0.17	0	100	100	27, 54, 88, 111	0
All	All	1600/1600 (100%)	-0.17	17 (1%)	77	78	18, 38, 73, 134	0

The worst 5 of 17 RSRZ outliers are listed below:

Mol	Chain	Res	Type	RSRZ
1	O	67	LYS	4.6
1	I	69	ARG	4.0
1	K	41	TYR	3.6
1	M	41	TYR	3.2
1	O	1	MSE	3.1

CONFIDENTIAL VALIDATION REPORT

6.2 Non-standard residues in protein, DNA, RNA chains ⓘ

In the following table, the Atoms column lists the number of modelled atoms in the group and the number defined in the chemical component dictionary. LLDF column lists the quality of electron density of the group with respect to its neighbouring residues in protein, DNA or RNA chains. The B-factors column lists the minimum, median, 95th percentile and maximum values of B factors of atoms in the group. The column labelled 'Q< 0.9' lists the number of atoms with occupancy less than 0.9.

Mol	Type	Chain	Res	Atoms	RSR	LLDF	B-factors($Å^2$)	Q<0.9
2	A23	L	45	25/26	0.13	1.50	24,29,39,50	0
2	A23	J	45	25/26	0.13	0.46	20,23,26,28	0
2	A23	D	45	25/26	0.13	0.07	18,21,24,28	0
2	A23	P	45	25/26	0.11	-0.07	23,27,31,34	0
2	A23	H	45	25/26	0.10	-0.51	24,25,28,29	0
2	A23	F	45	25/26	0.10	-0.60	20,23,27,27	0
2	A23	B	45	25/26	0.11	-0.80	40,47,53,58	0
2	A23	N	45	25/26	0.09	-0.86	27,32,36,40	0

6.3 Carbohydrates ⓘ

There are no carbohydrates in this entry.

6.4 Ligands ⓘ

In the following table, the Atoms column lists the number of modelled atoms in the group and the number defined in the chemical component dictionary. LLDF column lists the quality of electron density of the group with respect to its neighbouring residues in protein, DNA or RNA chains. The B-factors column lists the minimum, median, 95th percentile and maximum values of B factors of atoms in the group. The column labelled 'Q< 0.9' lists the number of atoms with occupancy less than 0.9.

Mol	Type	Chain	Res	Atoms	RSR	LLDF	B-factors($Å^2$)	Q<0.9
3	CA	J	104	1/1	0.39	29.83	110,110,110,110	0
3	CA	D	104	1/1	0.46	28.38	100,100,100,100	0
3	CA	N	104	1/1	0.39	17.22	72,72,72,72	0
3	CA	N	102	1/1	0.11	0.02	34,34,34,34	0
3	CA	H	101	1/1	0.09	-0.53	40,40,40,40	0
3	CA	K	201	1/1	0.09	-0.86	29,29,29,29	0
3	CA	L	103	1/1	0.10	-0.92	64,64,64,64	0
3	CA	B	101	1/1	0.09	-1.05	29,29,29,29	0
3	CA	L	106	1/1	0.10	-1.25	78,78,78,78	0
3	CA	P	102	1/1	0.08	-1.27	65,65,65,65	0

Continued on next page...

CONFIDENTIAL VALIDATION REPORT

Continued from previous page...

Mol	Type	Chain	Res	Atoms	RSR	LLDF	B-factors(Å^2)	Q<0.9
3	CA	F	101	1/1	0.08	-1.38	33,33,33,33	0
3	CA	B	104	1/1	0.08	-1.40	41,41,41,41	0
3	CA	P	103	1/1	0.10	-2.01	87,87,87,87	0
3	CA	F	103	1/1	0.07	-2.30	62,62,62,62	0
3	CA	P	101	1/1	0.07	-2.54	62,62,62,62	0
3	CA	F	102	1/1	0.06	-2.60	70,70,70,70	0
3	CA	F	105	1/1	0.07	-3.19	49,49,49,49	0
3	CA	H	107	1/1	0.07	-3.21	55,55,55,55	0
3	CA	D	107	1/1	0.04	-3.36	63,63,63,63	0
3	CA	P	104	1/1	0.07	-3.46	41,41,41,41	0
3	CA	H	103	1/1	0.06	-3.48	61,61,61,61	0
3	CA	H	105	1/1	0.04	-3.58	66,66,66,66	0
3	CA	G	201	1/1	0.04	-3.66	44,44,44,44	0
3	CA	L	101	1/1	0.07	-3.87	44,44,44,44	0
3	CA	L	102	1/1	0.06	-4.05	66,66,66,66	0
3	CA	J	103	1/1	0.04	-4.39	36,36,36,36	0
3	CA	B	102	1/1	0.05	-4.79	50,50,50,50	0
3	CA	D	102	1/1	0.04	-5.12	51,51,51,51	0
3	CA	F	104	1/1	0.04	-5.81	53,53,53,53	0
3	CA	J	106	1/1	0.08	-5.86	58,58,58,58	0
3	CA	B	103	1/1	0.07	-5.96	55,55,55,55	0
3	CA	N	105	1/1	0.05	-6.73	51,51,51,51	0
3	CA	N	106	1/1	0.05	-6.85	63,63,63,63	0
3	CA	B	106	1/1	0.04	-6.90	69,69,69,69	0
3	CA	L	105	1/1	0.05	-6.96	68,68,68,68	0
3	CA	D	103	1/1	0.05	-7.06	38,38,38,38	0
3	CA	N	101	1/1	0.05	-7.22	54,54,54,54	0
3	CA	D	106	1/1	0.05	-7.37	64,64,64,64	0
3	CA	L	104	1/1	0.05	-8.06	65,65,65,65	0
3	CA	N	103	1/1	0.06	-8.12	45,45,45,45	0
3	CA	J	102	1/1	0.06	-8.69	58,58,58,58	0
3	CA	H	104	1/1	0.04	-9.19	63,63,63,63	0
3	CA	J	105	1/1	0.09	-9.87	70,70,70,70	0
3	CA	H	102	1/1	0.03	-10.33	61,61,61,61	0
3	CA	D	105	1/1	0.04	-10.85	47,47,47,47	0
3	CA	B	105	1/1	0.03	-13.56	61,61,61,61	0
3	CA	D	101	1/1	0.04	-14.14	63,63,63,63	0
3	CA	J	101	1/1	0.05	-21.40	52,52,52,52	0
3	CA	H	106	1/1	0.04	-25.72	43,43,43,43	0

CONFIDENTIAL VALIDATION REPORT

6.5 Other polymers (i)

There are no such residues in this entry.

CONFIDENTIAL VALIDATION REPORT
